# Dynamics of severe accidents in the oil & gas energy sector derived from the authoritative ENergy-related severe accident database

**DOI:** 10.1371/journal.pone.0263962

**Published:** 2022-02-17

**Authors:** Arnaud Mignan, Matteo Spada, Peter Burgherr, Ziqi Wang, Didier Sornette

**Affiliations:** 1 Institute of Risk Analysis, Prediction and Management (Risks-X), Academy for Advanced Interdisciplinary Studies, Southern University of Science and Technology (SUSTech), Shenzhen, China; 2 Department of Earth and Space Sciences, Southern University of Science and Technology (SUSTech), Shenzhen, China; 3 Laboratory for Energy Systems Analysis, Paul Scherrer Institute (PSI), Villigen, Switzerland; 4 Institute of Sustainable Development, Zurich University of Applied Sciences (ZHAW), Winterthur, Switzerland; 5 Department of Civil and Environmental Engineering, University of California, Berkeley, California, United States of America; 6 Department of Management, Technology and Economics, Swiss Federal Institute of Technology (ETH) Zurich, Zurich, Switzerland; University of Naples Federico II, ITALY

## Abstract

Organized into a global network of critical infrastructures, the oil & gas industry remains to this day the main energy contributor to the world’s economy. Severe accidents occasionally occur resulting in fatalities and disruption. We build an oil & gas accident graph based on more than a thousand severe accidents for the period 1970–2016 recorded for refineries, tankers, and gas networks in the authoritative ENergy-related Severe Accident Database (ENSAD). We explore the distribution of potential chains-of-events leading to severe accidents by combining graph theory, Markov analysis and catastrophe dynamics. Using centrality measures, we first verify that human error is consistently the main source of accidents and that explosion, fire, toxic release, and element rupture are the principal sinks, but also the main catalysts for accident amplification. Second, we quantify the space of possible chains-of-events using the concept of fundamental matrix and rank them by defining a likelihood-based importance measure *γ*. We find that chains of up to five events can play a significant role in severe accidents, consisting of feedback loops of the aforementioned events but also of secondary events not directly identifiable from graph topology and yet participating in the most likely chains-of-events.

## Introduction

Global affairs have become increasingly complex, with intertwined networks known to be vulnerable to cascading failures [1, 2]. Triggers can be natural perils, such as earthquakes, cyclones, or floods [3, 4], or anthropogenic in nature, such as events related to global warming [5]. Of all existing lifeline networks, energy flows (especially of the oil & gas sector) are the most critical for the good functioning of our modern society. Major energy accidents often have large financial consequences, whose losses are combinations of lost product, repair costs, but also legal fees and fines by government agencies. For example, the 2005 BP Texas City refinery explosion killed 15 workers, injured 170, and led to financial losses of about $1.5 billion [6, 7]. Some of those events are natural-technological (or Natech) accidents: The 2011 Tohoku earthquake and associated great tsunami, mainly remembered for causing the failure of the Fukushima nuclear plant, also shut almost a third of the Japanese refining capacity. Accidents at critical infrastructures can lead to toxic material release, business interruption, and some economic slowdown [8]. The extensive flooding due to record rainfall during the 2017 Hurricane Harvey damaged more than 40 industrial plants and triggered over 100 toxic spillage events [9].

The study of energy accidents has a long history [10–15] and reflects a high energy security risk [16]. Of existing accident data inventories, the Major Energy Accidents (MEA) database contains 279 major energy accidents spanning over the course of the 20^th^ and early 21^st^ centuries [17]. As for the ENergy-related Severe Accident Database (ENSAD), it lists ~23,500 unique accident records between 1970 and 2016, of which 4,067 are considered severe (≥ 5 fatalities) [18, 19]. The two databases have been compared by Felder [12]. However, the dynamics of recorded accidents has yet to be better understood [20]. Energy models still generally do not allow for a deep exploration of extreme events and emergent endogenous phenomena [21], which consist of risk-amplifying cascading effects observed and already modelled in other complex systems [22–24].

Here, we explore the chains-of-events leading to severe accidents in the oil & gas industry using a data-driven approach based on ENSAD, which has been described as the most authoritative resource for comparative risk analysis of accidents in the energy sector [11, 13, 19]. This is the first study to apply graph theory and catastrophe dynamics modelling [25–28] to ENSAD, allowing us to describe the general topological properties of severe accidents at refineries [29], oil tankers [30] and gas networks [31], based on a rich database of more than a thousand events, spanning from 1970 to 2016, that includes information on the chains-of-events that led to these accidents. By describing severe accidents at critical infrastructures (CIs) with dependency risk graphs, we can first identify their main sources, catalysts, and sinks. Thanks to ENSAD’s volume of data, we can then go beyond existing studies [32, 33] by exploring interactions between 27 different events and offer a novel approach for the systematic, data-driven, exploration of events that amplify severe accidents. We use the concept of fundamental matrix from Markov chain theory recently introduced to catastrophe dynamics [34] to explore the full space of potential chains-of-events. We also define a likelihood-based importance measure to identify the most critical combinations of transitional events. Although Markov models are commonly used in process accident and cascading effect modelling, they are generally concerned with critical events within the chains [35] or with the final state [25], not with the dynamics of the chains themselves that might provide richer information for energy risk mitigation.

### The ENSAD database

We use the ENergy-related Severe Accident Database as sole input to our study [11, 13]. Severe accidents are defined in ENSAD as events leading to five or more fatalities, ten or more injuries, at least 200 evacuees, release of hydrocarbons exceeding 10,000 tons, enforced clean-up of land and water over areas of 25 km^2^ or greater, or an economic loss equal or greater to $5 million USD (inflation adjusted) [36]. Chains-of-events are extracted for the CIs part of the oil & gas sector. We considered 1,187 severe accidents in refineries (674), tankers (263), and natural gas networks (250). Offshore platforms for oil were not included for lack of readily available information on chains-of-accidents. However, the natural gas network includes the entire energy chain, from exploration and production, storage and distribution, and power plants. While the distribution of accidents is worldwide for refineries and gas networks, for tanker accidents, only those in the Mediterranean Sea were considered (one of the most active and accident-prone area in the world) [30].

One important attribute of ENSAD, which has so far not been analysed, is the description of the chain-of-events leading to a severe accident. The goal of this study is to remediate to this omission. Each chain-of-events is composed of 1 to 5 events, up to 6 for refineries in which case the initial trigger attribute is separate from the rest of the chain-of-events by construction. The number of possible events was originally 30 for refineries, 12 for tankers and 12 for gas networks, merged into 27 events for the 3 CI types combined. Chains-of-events here represent causality chains between events participating to a severe accident, not limited to loss-generating events. Successive (loss-generating) accidents are often referred to as cascading effects or domino effects [32]. Our study is more closely related to fault tree analysis [37] although we can distinguish between both types of events. Indeed, the sub-accidents that generate losses are all the events that can act as a sink in ENSAD (see below).

**Fig 1:**
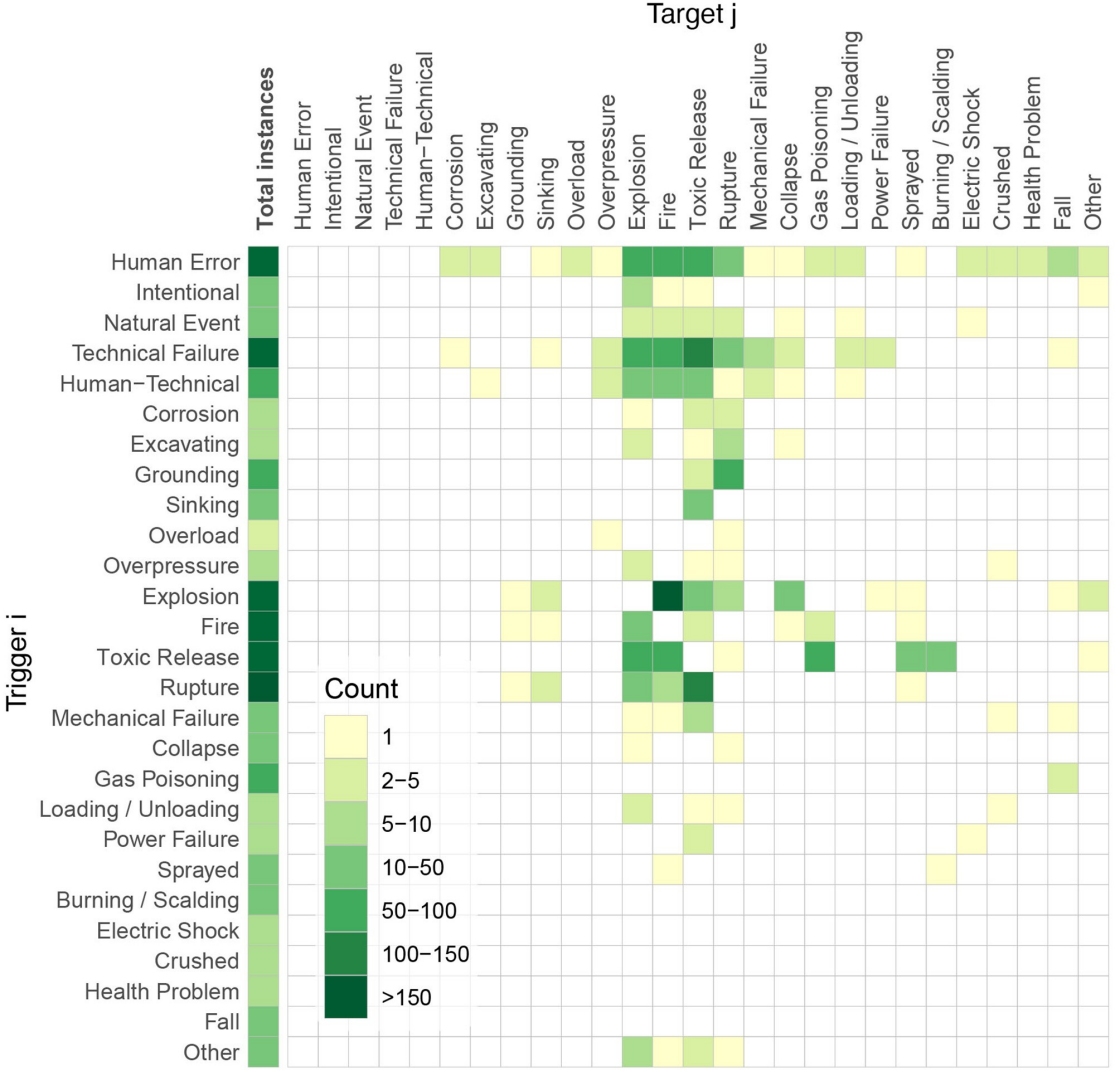
Event counts, total versus conditional in ENSAD for the oil & gas energy sector, for a total of 1,187 severe accidents. Considered critical infrastructures (CIs) are refineries, tankers, and gas networks.

In a preliminary step to chain-of-event encoding, we combined the events which were seldom observed in ENSAD into more representative categories for frequentist analysis. All natural-hazards (heavy rain, flood, landslide, earthquake, tsunami, lightning, (wind)storm, heatwave, and unknown natural triggers) were combined under the term Natural Event; machinery failure (1) and loss of power (1) were included in Technical Failure; personal health problem (3), radiation (1) and food poisoning (1) were combined in Health Problem; theft/scooping (1) (but not intentionally leading to an accident) and collision (37) were included in Human Error (if not already declared as Natural Event, e.g. due to fog). Several terms were also shortened to improve graph readability: ’Spill / Release / Leakage’ to Release (which can be toxic and/or inflammable), ’Rupture / Crack / Break Up’ to Rupture, ’Overflow / Overfitting / Overload’ to Overload, ’Asphyxiation / Gas Poisoning / Suffocating’ to Gas Poisoning, ’Sprayed / Sprinkled / Wetted’ to Sprayed, and ’Crushed / Pushed / Pinned’ to Crushed. It should be noted that ENSAD does not provide detailed causal factors for human errors, in contrast to the ones provided in the HFACS-OGI framework [38]. This is due to the lack of information retrievable for some accidents. Human error here implicitly includes violation of safety rules, violation of guidelines, management failure, *etc*. Fig 1 shows the total count *n*_*i*_ of cases per event type *i* as well as the count *n*_*ij*_ of one-to-one interactions, based on which the conditional probabilities to be used as input in section 3 are estimated with *p*_*ij*_ = *n*_*ij*_⁄*n*_*i*_. This frequentist approach provides a straightforward and informative, data-driven, inference.

It must be noted that our approach is subjected to the completeness, homogeneity, and uncertainties of ENSAD, as in any data-driven analysis. However, ENSAD has been shown to be more careful in its methodology compared to other available databases [12]. To avoid under-sampling, rare accident associations were preliminary merged into more representative categories, as described in the previous paragraph. The problem of heterogeneity between different CIs and the potential lack of comparability mentioned in the energy accident literature [12] is shown to be unfounded when considering chains-of-events attributes, as different oil & gas CIs share a number of hazardous elements. We will demonstrate below that refineries, tankers, and gas networks—despite their specificities—display similar accident network properties as when they are combined in one unique graph.

### Chain-of-event encoding & modelling

#### Graph definition

**Fig 2:**
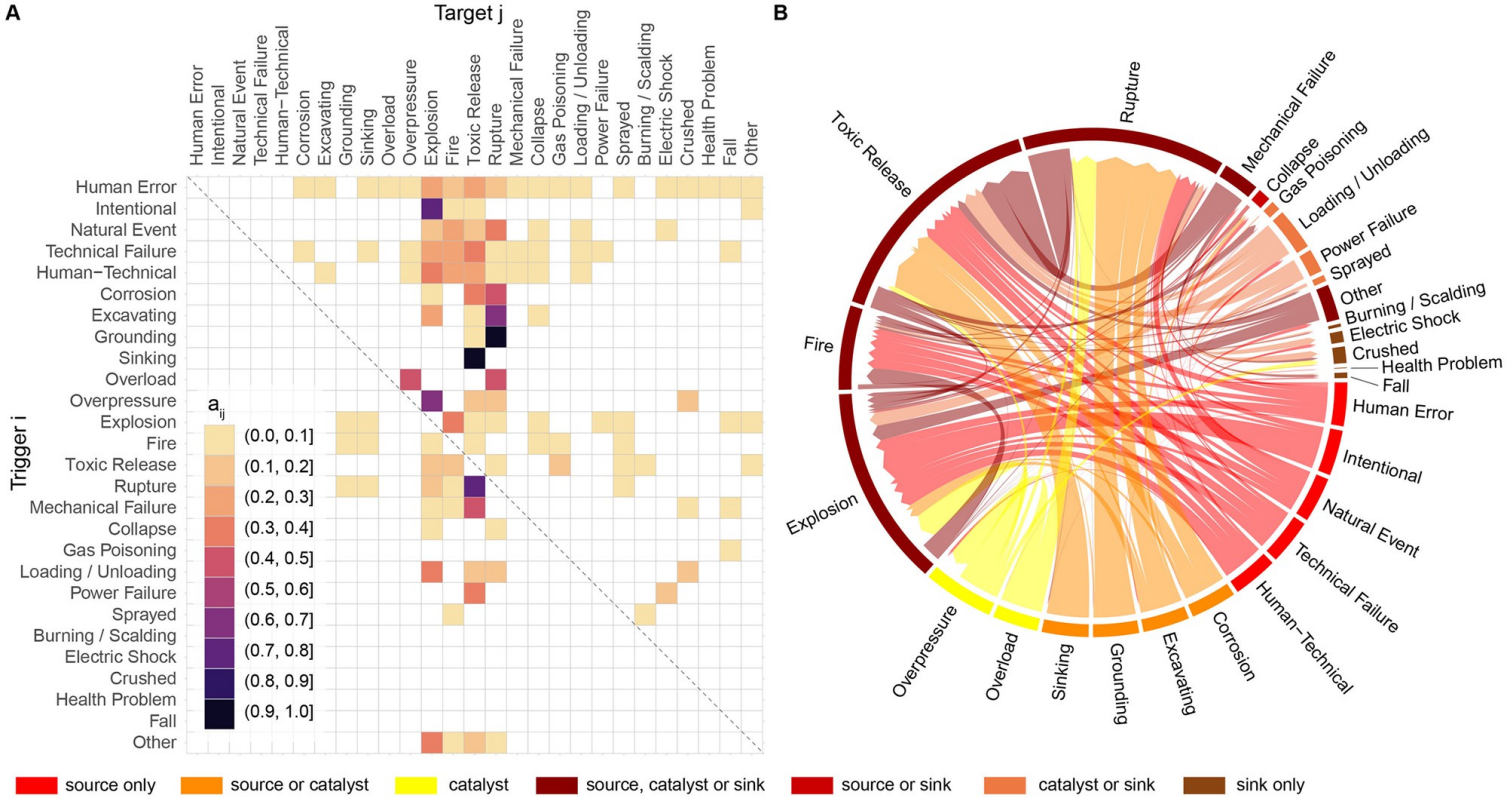
One-to-one interaction system leading to severe accidents in the energy sector. (A) Adjacency matrix ***A*** with one-to-one conditional frequentist probabilities derived from ENSAD (refineries, tankers, and gas networks combined); (B) Chord diagram representing the complexity of the directed chains-of-events, as encoded in the adjacency matrix. Note that sub-accidents (i.e., loss generating events) are all the events that can act as a sink in ENSAD.

**Fig 3:**
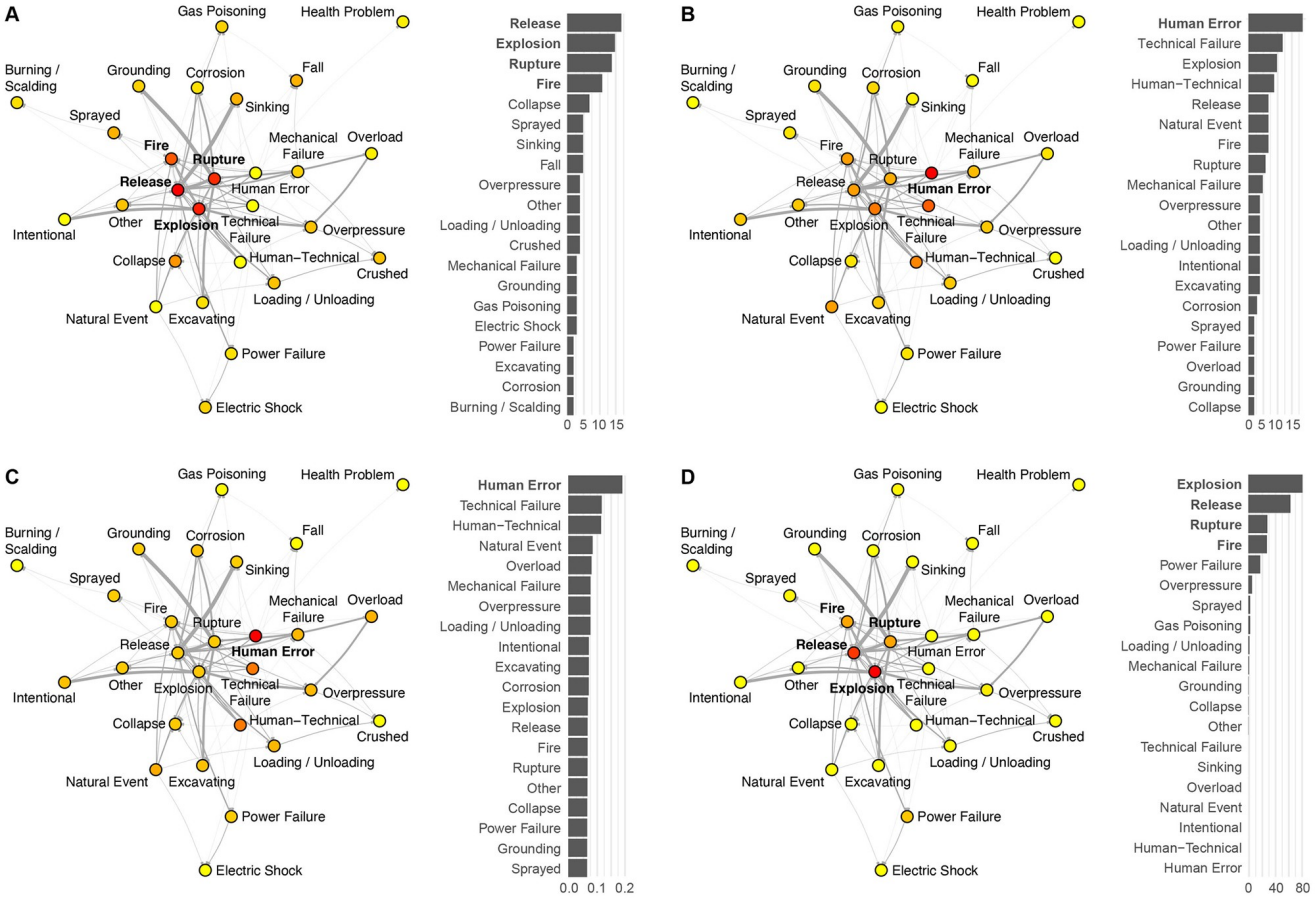
Centrality measures of the severe accident chains-of-events. (A) In-degree centrality; (B) out-degree centrality; (C) closeness centrality; (D) betweenness centrality. Influent nodes (or hazardous events) are highlighted in bold per centrality measure. Yellow-to-red colours of the vertices represent increasing centrality estimates, following the scales and ranking shown in the histograms.

As a first step in our analysis, we encode the chains-of-events given in ENSAD into a 27 × 27 adjacency matrix ***A*** where each element *a*_*ij*_ is the conditional frequentist probability of event *j* given the occurrence of event *i*. The result—derived from the data of Fig 1 —is shown here in Fig 2A. This accident interaction system is further represented by a chord diagram in Fig 2B to make the distinction between source events, catalysts and sink events, and by a directed weighted graph in Fig 3 for centrality measure analysis. We merged the causal networks of the different CI systems by assuming that similar CI elements play similar engineering roles at a generic level. This hypothesis will be shown to be reasonable in section 4 (with further plots given as S1 File).

We consider the three principal measures of centrality as explanatory metrics of the chains-of-events in severe accidents: degree (in- and out-degree), betweenness, and closeness [39]. Degree centrality of a vertex is the number of edges incident on the node; closeness centrality of a vertex is the average of the shortest path lengths from the vertex to all other vertices in the graph; betweenness centrality of a vertex is the number of the shortest paths that pass through that vertex. In catastrophe dynamics modelling [26–28], the main triggering events (or sources) are represented by high out-degree and closeness centralities. The main triggered events (or sinks) are represented by a high in-degree centrality. Catalysts of cascading effects are represented by a high betweenness centrality.

#### Markov chain theory

With severe accidents defined as the succession of individual events that interact with each other, we may assume that the dynamical process is described by the following linear differential equation

$$\frac{d\vec{X}(t)}{dt}=Q\vec{X}(t)$$ (1)

in which the dynamics of the vector $\vec{X}$ of events is described by the transition rate matrix ***Q*** that encodes all possible one-to-one direct interactions. The propagator for $\vec{X}(t)\to \vec{X}(t+\tau )$ can be written as ***P***(*τ*) = exp(***Q****τ*) with the entries *p*_*ij*_(*τ*) interpreted as the conditional probability of event *j* being triggered by event *i* for a period of *τ* and with ***A*** ≔ ***P***(*τ* = 1). We define an outflow event as a last (implicit) matrix entry for conservation of probabilities with conditional probability $1-\sum_{j=1}^{n}p_{ij}$. We also fix the last row of ***A*** as *p*_*n*+1_ = (0,…, 0, 1), i.e. the outflow event does not trigger any real event but itself, which is an absorbing state in Markov chain parlance. These 28^th^ column and row are not displayed on the adjacency matrix plots for convenience. As so, we do not directly apply Eq (1) but operate with the adjacency matrix, as shown in the next subsection.

Note that the proposed approach has two main limitations: first, the system dynamics considers one-to-one interactions (reflected in Eq 1) and not triplets or higher-order interactions although triggering may be caused or enhanced by the combination of multiple variables; second, the true temporal evolution of a chain-of-events is not modelled, only the ranks or succession of events with their cumulative count acting as an effective time. Eq 1 provides a first-order description of the process and the rate matrix, defined from the adjacency matrix, is directly encodable from ENSAD data. Including *n*-to-one interactions would require physical modelling which is out of the scope of the present empirical study. Moreover, the memorylessness property of Markov theory is a very common and well accepted assumption. Finally, the chains-of-events generated in the present study are implicitly assumed to occur in a natural physical time scale that is simplified into the consecutive trials in a Markov chain. Inclusion of common time intervals between different types of events would require details that are not available in the ENSAD.

#### Fundamental matrix definition

While the analysis of the matrix power ***A***^*τ*^ would suffice to explore the full space of possible interactions in a topological sense, we use the concept of fundamental matrix ***N*** to capture the frequency, or intensity, of each transition event before resting at the absorbing state:

$$N:={\left(I-A\right)}^{-1}=I+\sum_{\tau =1}^{\infty }A^{\tau }=I+A+A^{2}+\ldots =I+M$$ (2)

where the entry *n*_*ij*_ is the expected number of times the chain-of-events reaches event *j* given that the accident starts with event *i*; ***I*** is the identity matrix, *τ* the number of pseudo-time steps and ***M*** the interaction matrix [34], which excludes the step *τ* = 0. Eq 2 describes all possible combinations of chains-of-events via the matrix power ***A***^*τ*^ including amplification via the sum operator. Elements *m*_*ij*_(*τ*) are interpretable as the total set of scenarios leading to event *j* for a chain-of-events starting from event *i* and of length 1 to *τ*.

This study is the second application of the fundamental matrix to catastrophe dynamics. The first one, by Mignan and Wang [34], analysed the role of adjacency matrix topology on the matrix ***M***. It described the non-trivial behaviour of cascading patterns in terms of the space of possibilities covered, and of interaction amplification by feedback loops. That study further tested the approach on a small database of 29 historical catastrophes known for their domino effects. The present work is the first application of the fundamental matrix to the energy sector and to such a large database (more than one thousand accidents from ENSAD). Notice that the approach proposed earlier by Helbing and Kühnert [25] models the final state that is equivalent to ***A***^*τ*^, but based on the rate matrix ***Q***. In contrast, the interaction ***M*** provides some information on all the transition states of the chain.

#### Importance measure definition

For the purpose of risk mitigation, an importance measure of a chain of *m* events ***Z***(*m*) = (*z*_1_, *z*_2_, …, *z*_*m*_) is defined using the Markov property. It is derived from the likelihood function

$$L(z_{1},z_{2},\ldots ,z_{m})=\prod_{i=1}^{m}p(z_{i}|z_{i-1})$$ (3)

where we set *p*(*z*_1_|*z*_0_) ≡ *p*(*z*_1_). The conditional probability can be directly read from ***A***, i.e. *p*(*z*_*i*_|*z*_*i*−1_) = *a*(*z*_*i*−1_, *z*_*i*_). Since any chain that has not yet reached the absorbing state has the potential to further grow, it is useful to define the concept of a *complete chain* as any chain that rests at the absorbing state. Formally, let the absorbing state be denoted by *z*_*a*_, a chain (*z*_1_, *z*_2_,…, *z*_*m*_) is said to be complete iff *z*_*m*_ = *z*_*a*_, and *z*_*i*_ ≠ *z*_*a*_ for *i* ∈ {1, 2,…, *m* − 1}. An importance measure for a complete chain with *m* events can then be defined as:

$$\gamma (z_{1},\ldots ,z_{m-1},z_{a})=\frac{L(z_{1},\ldots ,z_{m-1},z_{a})}{\sum_{Z^{'}(m)\in \sigma_{z}(m)}L\left(Z^{'}(m)\right)}=\frac{\prod_{i=1}^{a}a\{z_{i-1},z_{i}\}}{\sum_{Z^{'}(m)\in \sigma_{z}(m)}L\left(Z^{'}(m)\right)}$$ (4)

where L is the likelihood function, *z*_*m*_ = *z*_*a*_ the absorbing (final) state, and *σ*_*z*_(*m*) the set of all possible complete chains with *m* events. Note that *γ* can be interpreted as the probability of a specific chain among chains-of-*m*-events. It can thus be used for severe accident ranking.

### ENSAD application

The ENSAD accident interaction encoding is first described in Figs 1 and 2. Counts of one-to-one interactions go from one instance for the rarest interactions in ENSAD to more than 150 in the case of the most common triggering process, which is an explosion triggering a fire (Fig 1). We observe a rich variety of 117 potential one-to-one interactions. Since most accidents considered in ENSAD are described as chains-of-events, the conditional frequentist probabilities *a*_*ij*_ can be relatively high, with 36% of cases above 0.1 (Fig 2). Two interactions have a probability greater than 0.9 of occurring, which are grounding (i.e., impact to seabed or waterway side, also known as stranding) to rupture, and tanker sinking to toxic material release. All interactions are constrained by the underlying assumption that an accident is severe in ENSAD. Hence, a tanker sinking implicitly means that a toxic spill follows for the accident to be severe. The same rule applies for all other interactions.

The ENSAD already contains some examples of long historical chains-of-events [19]. For example, in the case of refineries, one of the worst industrial disasters in recent U.S. history happened on the 23^rd^ of March 2005. On that day, at the BP Texas City Refinery (USA), due to an error, a raffinate splitter tower has been overfilled. Once the isomerization (ISOM) unit was started, the overfill caused the opening of the pressure relief devices, which resulted in a flammable liquid geyser from a blowdown stack that was not equipped with a flare. The release of flammables led to an explosion followed by fires. The latter killed 15 workers located close to the source of the accidents and injured another 180, alarmed the community (with 43,000 people ordered to remain indoors), and resulted in financial losses exceeding $1.5 billion, due to the damages to the refinery and the houses in a radius of 750 meters from it. Other examples include (*i*) a release followed by fire from a depropanizer, which caused the injury of a worker at the Alkylation unit of the Delaware City refinery (USA) in 2015, (*ii*) an explosion followed by fire, which caused the injury of 6 workers during maintenance of a tank at the Aspropyrgos refinery in Greece in 2015.

In the case of gas networks, an example taken from ENSAD is the accident that happened 50 km south-west from Brussels (Belgium) in 2004. In an excavation process during a construction work, a scraper ruptured a major underground high-pressure natural gas pipeline. Immediately, fire fighters and police rushed to secure the area, but an explosion occurred followed by fire, which killed 17 persons and injured 120. Other examples are the explosion of a pipeline caused by corrosion and the consequent release of natural gas, followed by fires that killed 22 people and injured 40 in India in 2014; the explosion of a CNG station caused by an intentional attack, which caused 25 fatalities and 100 injuries in Pakistan in 2011. Some of those chains-of-events are not limited to gas networks. Some events are however associated to CI-specific elements, such as excavation when pipelines are involved.

For tankers, one of the worst accidents in the Mediterranean Sea happened near the port of Genoa in 1991. The Cypriot oil tanker Haven, anchored off Genoa, loaded with 144,000 tons of crude oil, caught fire, exploded, and broke into three parts. One of them sank on the spot, the other parts were taken hold of and sank during their towing, releasing the entire loaded crude oil in the sea. In addition, the accident caused 6 fatalities and 30 injuries among the people on the tanker. Other examples are the Ottoman Integrity tanker, which released 3300 tons of crude oil during shipping in the Peloponnesus area in Greece in 2015; a tanker grounded at the port of Tarragona (Spain), which sank and released the remaining crude oil in the vessel (around 25 tons) in 2008. All those examples were encoded as one-to-one interactions in the previous section but can be retrieved via Markov theory.

Here, we generalise the process of interactions by applying the above theory to the ENSAD. Based on the adjacency matrix shown in Fig 2, centrality measures are computed and plotted on the matching graphs in Fig 3. They are then ranked in histograms to identify the main sources, sinks and catalysts of severe accidents. The main triggers (or sources) are found to be human error followed by technical failure as they have the highest out-degree and closeness centralities. We also observe that the main triggered events (or sinks), represented by a high in-degree centrality, are toxic (including inflammable) release, explosion, element rupture and fire. These events are also the four main catalysts for cascading effects, being also represented by the highest betweenness centralities. These results are consistent across CI types, supporting our approach to combine all CIs in one graph. Figures similar to Fig 3 but for the individuals CIs are given as S1 File. S1 Fig in S1 File shows the accident network and matching centrality measures for refineries. Results are very similar to the combined oil & gas CI network since this class is dominating the full dataset with 57% of cases. S2 Fig in S1 File shows the same plots for tankers. For this CI, sinking replaces explosion in the top sinks (highest in-degree centrality), the other sinks remaining the same (release, rupture, fire). Human error remains the dominant initial trigger, the only event being at the highest rank for both out-degree and closeness centralities. Similar catalysts are found except again for sinking replacing explosion (highest betweenness centrality). S3 Fig in S1 File, finally, shows the results for gas networks. The accident network topology is similar to the one for refineries and all oil & gas CIs combined. Quantified by a globally comprehensive data source, our results confirm the existing literature that most accidents are due to human errors [38, 40–42] but also that fire, explosion, and toxic release are the major hazards in the industry [43, 44], as well as the main sources for further domino effects [32].

**Fig 4:**
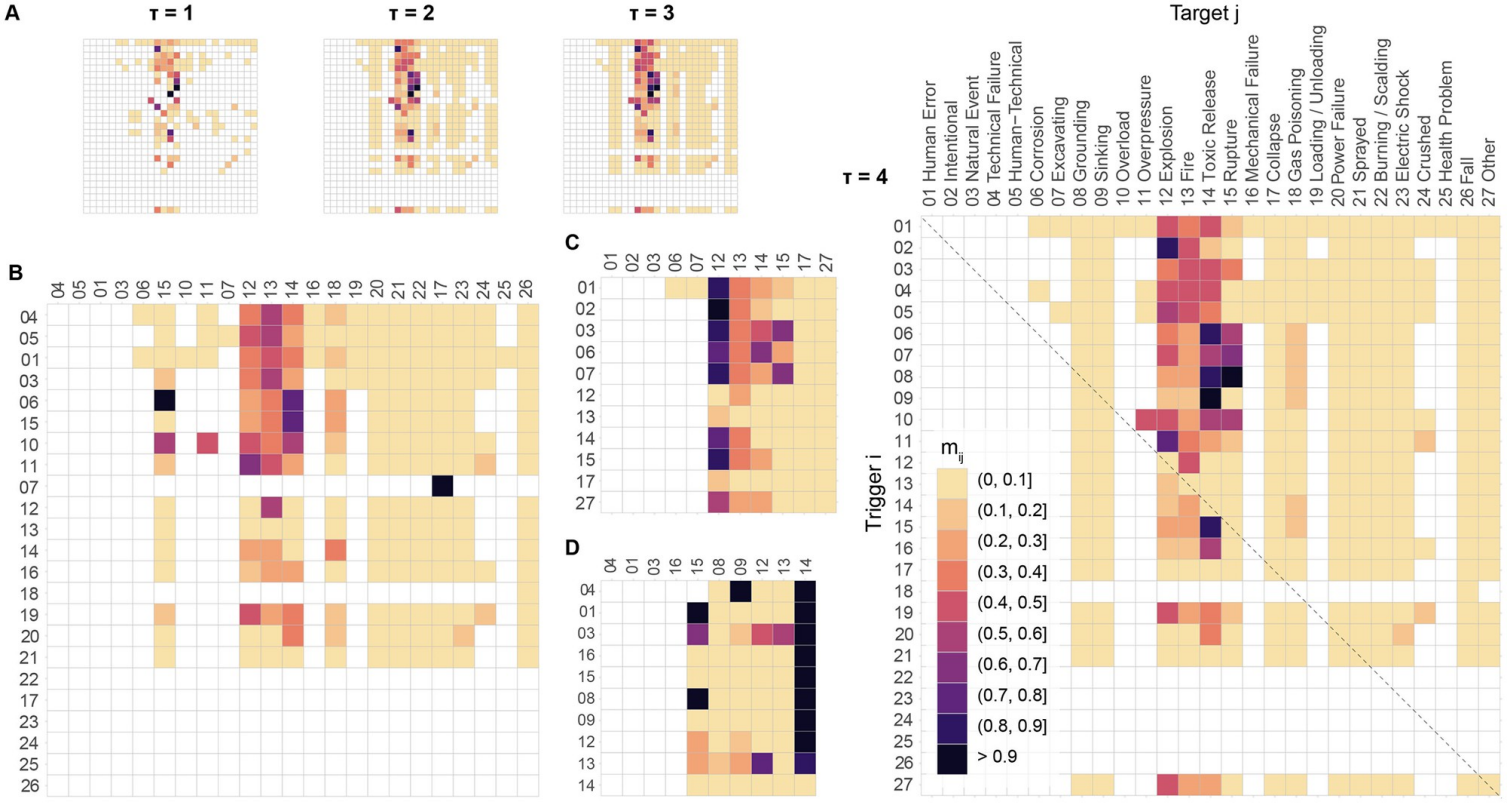
Interaction matrix *M* encoding chains-of-events of length up to *τ*. (A) All CIs combined with ***M*** evolving from *τ* = 1 (i.e., adjacency matrix ***A***) to *τ* = 4. (B) ***M***(*τ* = 4) for refineries. (C) ***M***(*τ* = 4) for gas networks. (D) ***M***(*τ* = 4) for tankers.

To go beyond the identification and ranking of individual critical events by centrality measures, we investigate the potential amplification of chains-of-events via the interaction matrix ***M*** previously defined (Eq 2) and plotted in Fig 4. We are here concerned with event *j* being a sub-accident, meaning a loss-generating sink event, as defined by the colour scheme in Fig 2B. We find that the dynamical process stabilises rapidly, around *τ* = 4, and expands the space of possibilities significantly compared to direct interactions *a*_*ij*_ = *m*_*ij*_ (*τ* = 1). This is captured in Fig 4A in which we see the number of possible interactions (i.e., non-zero cells) increase at each step to then remain constant at *τ* = 4. The values *m*_*ij*_ can still evolve at this stage but changes are dwarfed relative to the summation of the previous steps, which is due to the fact that the matrix power ***A***^*τ*^ tends to zero for increasing *τ*. Of all trigger events (27 minus 5 sink-only) and triggerable events (27 minus 5 source-only), 64% (14/22) of triggerable events can be triggered, directly or indirectly, by 95% (21/22) of trigger events. This suggests the potential for surprising accidents that were never experienced before. Longer chains-of-events (*τ* > 4) can be considered unsignificant as their contribution, terms ***A***^*τ*^ for *τ* > 4 in Eq 2, becomes negligible. The interaction matrix ***M*** also shows which accidents *j* are most prone to occur due to a multiplicity of chains-of-events, turning towards purple-to-black as *τ* increases. Those events are again fire, explosion, toxic release, and rupture, which is consistent with our previous measures of high betweenness and in-degree centralities. Those patterns are more pronounced when Eq 2 is applied to individual CIs as shown in Fig 4B–4D for refineries, gas networks and tankers, respectively. This is due to the smaller number of instances leading to some high conditional probabilities at *τ* = 1 in ENSAD. Those results remain consistent with the measures of high betweenness and in-degree centralities described in the S1 File. However, it shows that merging all CIs in one graph yields more stable patterns.

**Fig 5:**
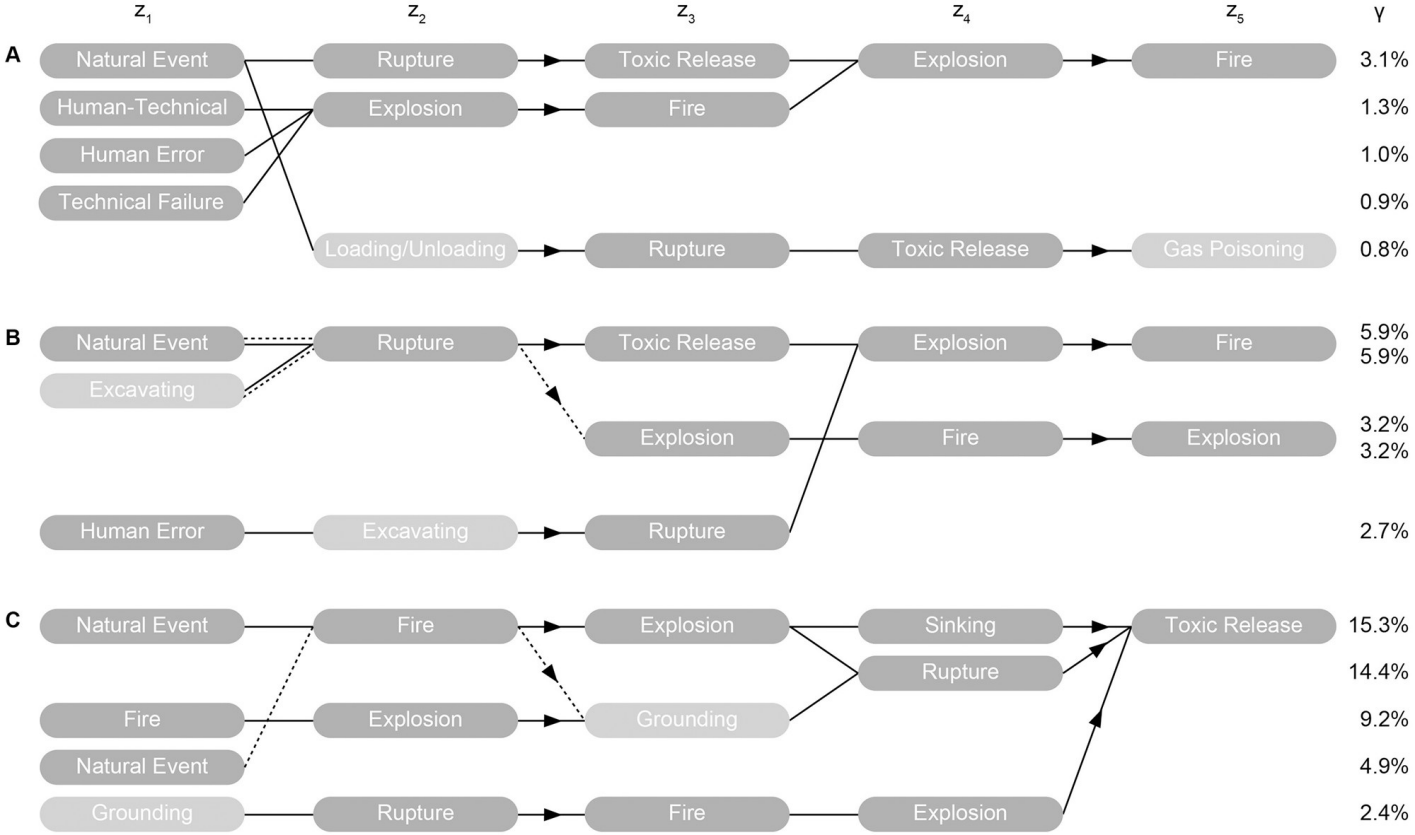
Ranking of chains-of-5-events from the importance measure *γ* for different oil & gas CIs. (A) Refinery case. (B) Gas network case. (C) Tanker case. Secondary events that were not highly ranked from centrality measures are represented in light grey.

Chains-of-*m* -events with *m* = 5 (i.e. *τ* = 4) are finally ranked per CI by Eq 4, with the respective top-5 chains shown in Fig 5. We do not show smaller chains but recall that they are also included in ***M***. We chose *τ* = 4 based on the results presented above. The approach provides a simple way to systematically explore emergent chains-of-events, including feedback loops. The best example observed here is the chain *explosion* → *fire* → *explosion* → *fire*. While the four critical events (explosion, fire, release, and rupture) drive the domino effects (shown in dark grey), we see other events, such as loading/unloading, gas poisoning, excavating or grounding, participate to the emergence of such chains (shown in light grey). Note that more would be found by analysing lower-*γ* scenarios. Such events were only secondary in terms of centrality measures and their relative important may have been missed if solely using graph theory (see Fig 3). The metric *γ*, being interpretable as the probability of a specific chain among chains-of-*m*-events, allows prioritizing mitigation measures not only based on critical events but on critical chains-of-events that contain more information. This is exemplified by the ENSAD example in Fig 5.

### Concluding remarks

Severe accidents in the oil & gas industry can have important repercussions on the global energy flow, and thus on the economy. Due to the complexity of the critical infrastructures involved, those accidents generally result from complex chains-of-events. The critical events that participate to the build-up to catastrophe (such as human error, explosion, fire) are well-known and identifiable from graph theory. However, the systematic exploration of plausible chains-of-events must provide richer information for risk mitigation. This was feasible in the present study via the analysis of the authoritative ENergy-related Severe Accident Database. We used the concept of fundamental matrix and defined an importance measure to identify the complexity of interactions and rank emergent behaviours, respectively.

The topological analysis of more than a thousand oil & gas accidents from the ENSAD confirmed the critical role of fire, explosion, and toxic release (but also element rupture) in chains-of-events leading to severe accidents, as well as human error as being the main source of such accidents, globally. Our dynamical analysis further demonstrated the role of long chains-of-events (up to 5 events), the significant extension of the space of possible indirect interactions, the feedback loops (*fire* ↔ *explosion*) and the role of secondary events on top of the four critical events (fire, explosion, release, rupture) in the most likely chains-of-events. This complexity was modelled via the interaction matrix ***M*** and ranked via the importance measure *γ*. Although the method is applicable to any adjacency matrix ***A*** of one-to-one interactions, only large databases such as ENSAD can lead to its full appreciation. Considering CIs individually, which reduced the number of instances, showed the important role of sample size. Interestingly, the full exploration of potential chains-of-events based on a narrower set of observed chains-of-events is one approach to model downward counterfactuals [45].

These insights should inform the optimal allocation of resources and needed level of maintenance in oil & gas CIs. With CI risk management crucially dependent on the analysis of causes and effects of failures [20, 23, 46, 47], especially of the evaluation of the entire accident sequence [33], decision-makers and first responders alike could use the ENSAD-based adjacency and interaction matrices to estimate the *a priori* cascading risk for the stress-testing of their CIs [48]. A Bayesian approach could be applied to update the ENSAD prior with CI-specific characteristics [4, 49]. Future improvements in accident databases shall further improve this approach. In general, we believe that data-driven dynamics will help minimize the impact of surprising accidents in the energy sector.

## Supporting information

S1 File Centrality measures of the severe accident chains-of-events at different types of critical infrastructures. Refineries (S1 Fig), tankers (S2 Fig) and gas networks (S3 Fig).
(PDF)
