# Supplementary material for: Dynamics of severe accidents in the oil & gas energy sector derived from the authoritative ENergy-related severe accident database
Source: PLoS One. 2022 Feb 17;17(2):e0263962. doi: 10.1371/journal.pone.0263962 (PMC8853565; doi:10.1371/journal.pone.0263962)
Supplement: S1 File — Refineries (S1 Fig), tankers (S2 Fig) and gas networks (S3 Fig). (PDF) [file pone.0263962.s001.pdf]

SUPPORTING INFORMATION

Dynamics of Severe Accidents in the Oil & Gas Energy Sector Derived from the Authoritative ENergy-related Severe Accident Database

Arnaud Mignan[1,2,3,*], Matteo Spada[4,3], Peter Burgherr[3], Ziqi Wang[5], Didier Sornette[1,6]

1 Institute of Risk Analysis, Prediction and Management (Risks-X), Academy for Advanced Interdisciplinary Studies, Southern University of Science and Technology (SUSTech), Shenzhen, China
2 Department of Earth and Space Sciences, Southern University of Science and Technology (SUSTech), Shenzhen, China
3 Laboratory for Energy Systems Analysis, Paul Scherrer Institute (PSI), Villigen PSI, Switzerland
4 Institute of Sustainable Development, Zurich University of Applied Sciences (ZHAW), Winterthur, Switzerland
5 Department of Civil and Environmental Engineering, University of California, Berkeley, USA
6 Department of Management, Technology and Economics, Swiss Federal Institute of Technology (ETH) Zurich, Switzerland

* mignana@sustech.edu.cn

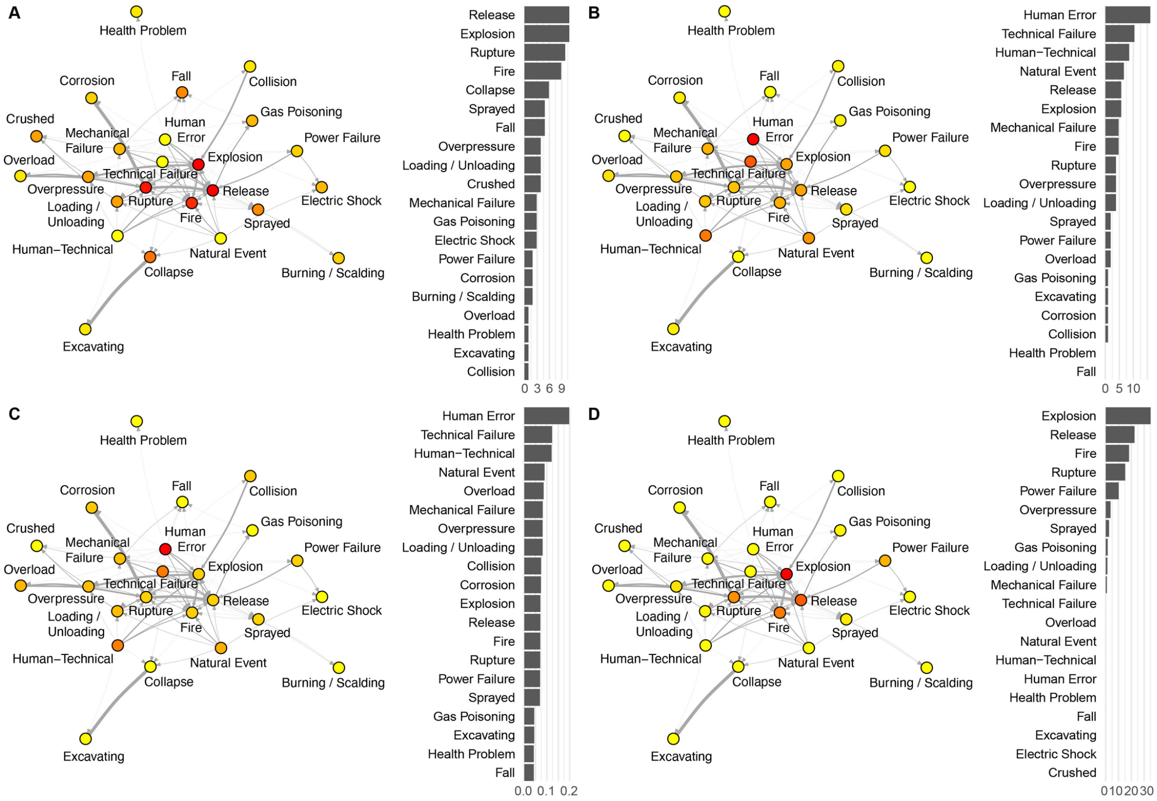

S1 Figure. Centrality measures of the severe accident chains-of-events at refineries. (A) In-degree centrality; (B) Out-degree centrality; (C) Closeness centrality; (D) Betweenness centrality. Yellow-to-red colours of the vertices represent increasing centrality estimates, following the ranking shown in the histograms.

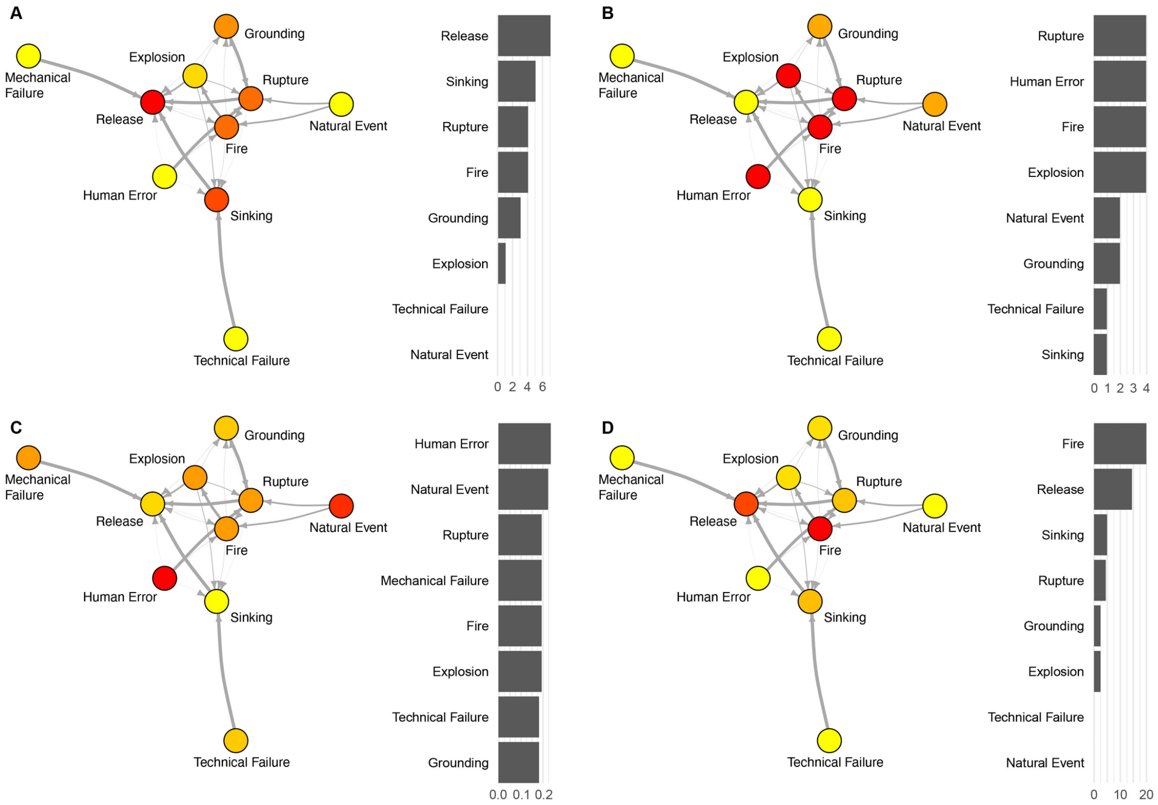

S2 Figure. Centrality measures of the severe accident chains-of-events at tankers. (A) In-degree centrality; (B) Out-degree centrality; (C) Closeness centrality; (D) Betweenness centrality. Yellow-to-red colours of the vertices represent increasing centrality estimates, following the ranking shown in the histograms.

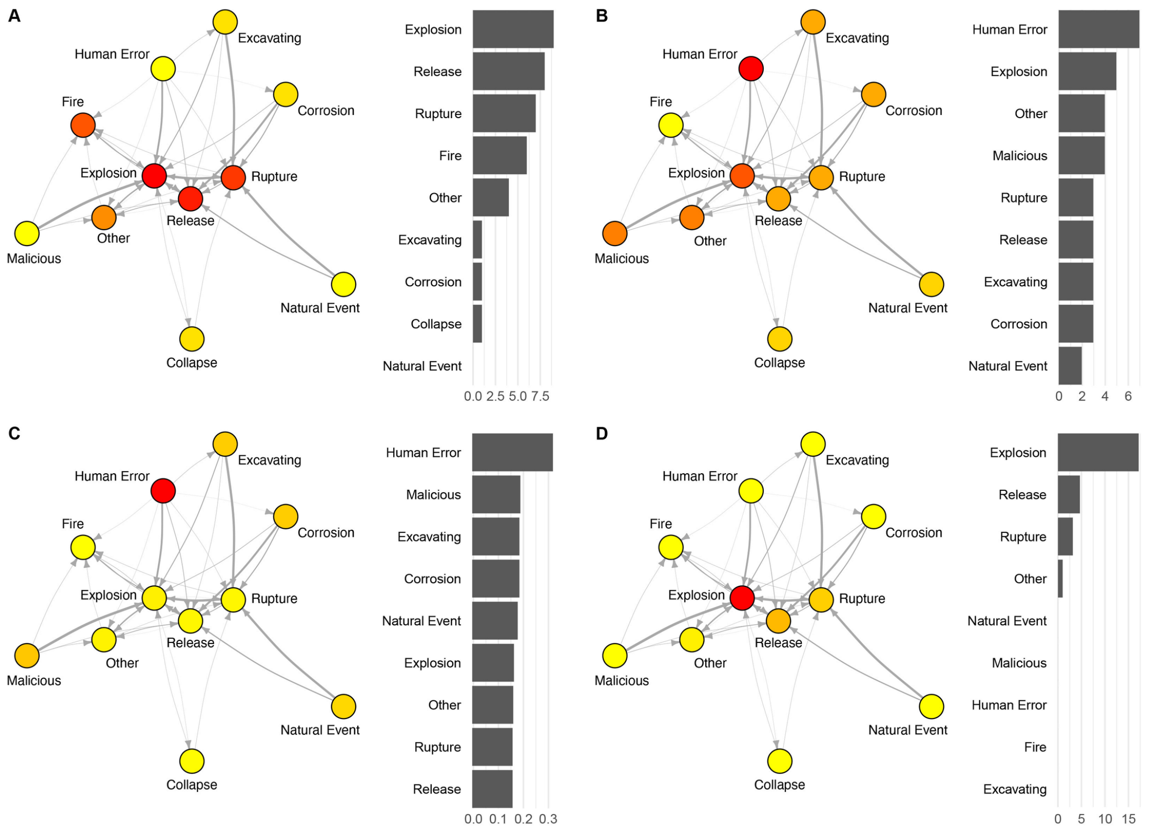

S3 Figure. Centrality measures of the severe accident chains-of-events at gas networks. (A) In-degree centrality; (B) Out-degree centrality; (C) Closeness centrality; (D) Betweenness centrality. Yellow-to-red colours of the vertices represent increasing centrality estimates, following the ranking shown in the histograms.
